# Variation in pre- and post-copulatory sexual selection on male genital size in two species of lygaeid bug

**DOI:** 10.1007/s00265-016-2082-6

**Published:** 2016-03-02

**Authors:** Liam R. Dougherty, David M. Shuker

**Affiliations:** School of Biology, University of St Andrews, St Andrews, KY16 9TH UK; Centre for Evolutionary Biology, School of Animal Biology, The University of Western Australia, Crawley, WA 6009 Australia

**Keywords:** Genital evolution, Sexual selection, Social environment, Lygaeinae, Post-copulatory, Pre-copulatory, Meta-analysis

## Abstract

**Abstract:**

Sexual selection has been shown to be the driving force behind the evolution of the sometimes extreme and elaborate genitalia of many species. Sexual selection may arise before and/or after mating, or vary according to other factors such as the social environment. However, bouts of selection are typically considered in isolation. We measured the strength and pattern of selection acting on the length of the male intromittent organ (or processus) in two closely related species of lygaeid seed bug: *Lygaeus equestris* and *Lygaeus simulans*. In both species, we measured both pre- and post-copulatory selection. For *L. equestris*, we also varied the experimental choice design used in mating trials. We found contrasting pre- and post-copulatory selection on processus length in *L. equestris*. Furthermore, significant pre-copulatory selection was only seen in mating trials in which two males were present. This selection likely arises indirectly due to selection on a correlated trait, as the processus does not interact with the female prior to copulation. In contrast, we were unable to detect significant pre- or post-copulatory selection on processus length in *L. simulans*. However, a formal meta-analysis of previous estimates of post-copulatory selection on processus length in *L. simulans* suggests that there is significant stabilising selection across studies, but the strength of selection varies between experiments. Our results emphasise that the strength and direction of sexual selection on genital traits may be multifaceted and can vary across studies, social contexts and different stages of reproduction.

**Significance statement:**

Animal genitalia vary greatly in size and complexity across species, and selection acting on genital size and shape can be complex. In this study, we show that the length of the penis in two species of seed bug is subject to complex patterns of selection, varying depending on the social context and whether selection is measured before or after mating. In one of the species, we show unexpectedly that penis length is correlated with male mating success, despite the fact that the penis does not interact with the female prior to mating. Our results highlight the fact that genitalia may be subject to both direct and indirect selection at different stages of mating and that to fully understand the evolution of such traits we should combine estimates of selection arising from these multiple episodes.

**Electronic supplementary material:**

The online version of this article (doi:10.1007/s00265-016-2082-6) contains supplementary material, which is available to authorized users.

## Introduction

Genitalia show an extraordinary amount of morphological variation across the animal kingdom and may vary even amongst very closely related species (Eberhard 1985; Hosken and Stockley 2004). The most general explanation for this diversity appears to be sexual selection (Eberhard 1985; Arnqvist 1997; Hosken and Stockley 2004; Simmons 2014). This is supported by correlational and manipulative studies that have found a significant relationship between genital morphology and reproductive success in several species (Simmons 2014). Importantly, it is well-known that sexual selection on genital morphology can act both prior to copulation by influencing mating success or post-copulation by influencing sperm transfer and fertilisation success (Hosken and Stockley 2004; Simmons 2014). In some cases, a single genital trait may influence both pre- and post-copulatory reproductive success (e.g. LeVasseur‐Viens et al. 2015; Frazee and Masly 2015). Despite this, these episodes of selection are traditionally considered in isolation, in studies of both genital and non-genital traits (Hunt et al. 2009; Kvarnemo and Simmons 2013). Modern statistical methods (such as the regression-based approach of Lande and Arnold 1983) allow us to determine both the shape and the strength of selection on a given trait (Kingsolver et al. 2001; 2012; Morrissey and Sakrejda 2013). By measuring selection, we can gain insights into which aspects of the physical or social environment are driving current selection, and we can also make predictions about the future evolutionary trajectory a trait may take. These methods allow us to estimate the total selection acting on a trait, which can be separated into selection acting directly on the trait of interest, and selection acting indirectly on the trait via selection on one or more correlated (and potential unmeasured) traits (Kingsolver et al. 2012).

The strength and shape of sexual selection acting on phenotypic traits may vary with the social environment (Miller and Svensson 2014). For example, if competition for mates is high, then mating success may depend on the number of rivals or potential mating partners that are available. Studies examining the strength of mating preferences in animals may vary the ‘choice design’, which is simply the number of mate options a subject is presented with (Wagner 1998). Studies can use either a no-choice test, in which only a single option is presented to a subject, or a choice test, in which multiple options (usually two) are presented. Experimental design has recently been shown to have a strong effect on the strength of mate choice across species, with choice being significantly stronger when tested using multiple-choice tests compared to no-choice tests (Dougherty and Shuker 2015a). Additionally, if sexual selection primarily arises via intrasexual competition, for example in the case of selection on weapons used in contests for access to mates (Emlen 2008), then this selection will be absent when no rivals are present but should be detectable when rivals are present. These factors could also influence post-copulatory sexual selection, for example if males allocate ejaculates differently depending on the number of potential rivals (Kelly and Jennions 2011).

In some insects, the male intromittent organ ends in an extremely thin, elongate tube sometimes referred to as a flagellum. This trait is seen for example in Coleoptera (Rodriguez 1995; Rodriguez et al. 2004; Gack and Peschke 2005), Hemiptera (Tadler 1999) and Dermaptera (Kamimura 2005; van Lieshout and Elgar 2011). Sexual selection has been suggested to be the primary mechanism driving the evolution of elongate genitalia (Eberhard 1985). However, very few studies have investigated the strength of selection on extremely elongate genitalia in insects (though see Rodriguez 1995; Rodriguez et al. 2004).

**Fig. 1:**
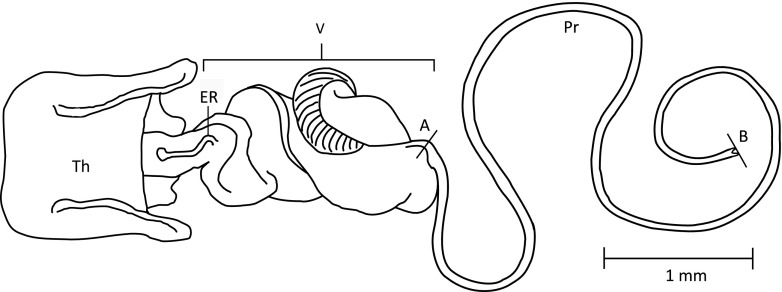
Drawing of the basic anatomy of the intromittent organ of *L. equestris* and *L. simulans. Th* theca, *V* vesica, *ER* ejaculatory reservoir, *Pr* processus

In this study, we measure selection acting on the length of the intromittent organ in the seed bug *Lygaeus equestris* and its sister species *L. simulans*. In both species, the intromittent organ ends in a long, coiled *processus gonopori* (hereafter referred to as processus for short), which is around two-thirds of a male’s body length (Ludwig 1926; Sillén-Tullberg 1981; Fig. 1). These two species are very closely related and morphologically very similar and were only described as separate species relatively recently (Deckert 1985). However, they can be reliably distinguished based on differences in the shape of the male genital claspers (Deckert 1985) and also in the length of the processus (see below). Significant differences in genital morphology between closely related species suggest that genital evolution has occurred relatively rapidly. The extreme length of the processus in both species also suggests that sexual selection may have played a role in its evolution. Indeed, previous work in *L. simulans* has found evidence for significant stabilising, post-copulatory selection on male processus length (Tadler 1999). Additionally, the relatively simple nature of the processus means that its size is easily and accurately quantified.

We assessed how the strength and pattern of selection on processus length varied between different stages of mating (before or after mating), social contexts (the number of males and females present during mating trials) and studies. We present the results of two experiments. In the first, we correlated processus length with male reproductive success using a sample of *L. equestris* males used in a previous experiment in which mating trials were performed using four experimental choice designs (Dougherty and Shuker 2014). We then performed a second experiment in *L. simulans* in which male reproductive success was recorded in no-choice mating trials. We expand on the earlier study by Tadler (1999) by also recording male mating success. For both species, we thus compared the strength and pattern of selection on processus length both before and during/after mating. We also attempted to combine these episodes of selection by examining the post-copulatory success of all males (including those that did not mate), giving an estimate of the overall selection acting on processus length. For *L. equestris*, we also assessed whether the choice design had a significant effect on the strength of selection. Finally, we assessed how the strength of selection varied across studies in *L. simulans* by using formal meta-analysis to combine selection gradients reported in previous studies with those found here.

## Methods

### Study species and insect husbandry

*Lygaeus equestris* L. and *L. simulans* Deckert are two closely related species of lygaeid bug (Heteroptera: Lygaeiodea; Lygaeinae). Both species are behaviourally and morphologically very similar. Males and females mate multiply during the breeding season (Solbreck 1972; Sillén-Tullberg 1981), and copulation duration is highly variable but may last in excess of 16 h (Sillén-Tullberg 1981; Shuker et al. 2006). In both species, fertilisation is very likely after 3–6 h of mating (Sillén-Tullberg 1981; Micholitsch et al. 2000), and so very long copulations probably serve as a form of post-copulatory mate-guarding, with males acting as a ‘living mating plug’ (Sillén-Tullberg 1981). There is no obvious courtship prior to mating in either species; instead, the male typically mounts the female in an attempt to achieve intromission (Sillén-Tullberg 1981; Tadler 1999). Females typically attempt to resist any male mating attempts, so that any mate choice probably results from a sexual conflict over mating (Shuker et al. 2006). In both species, males possess a pair of external genital claspers which are used first to grab the female and hold her in place, and second to unfold the ovipositor so that the aedeagus can be inserted (Deckert 1985; Tadler 1999). Once the aedeagus is inserted the claspers lock in place (Dougherty et al. 2015), and the pair moves to the characteristic ‘end-to-end’ mating position (Sillén-Tullberg 1981). During mating, females are frequently seen to kick males and rock from side to side, seemingly in an attempt to cause the male to detach (Sillén-Tullberg 1981). However, such behaviour may be observed for several hours with no sign of copulation ending (LRD pers. obs.). Therefore, it seems that female resistance behaviour may be effective at preventing unwanted matings, but not at influencing copulation duration once the male claspers are locked in. Finally, previous studies have shown that there is pre-copulatory selection on male and female body size in *L. equestris* (Burdfield-Steel et al. 2013; Dougherty and Shuker 2014); however, this is not influenced by the number of males present (Dougherty and Shuker 2014).

The male intromittent organ is almost identical in both species, with the exception of its length (see below). It consists of two distinct parts (Fig. 1): a soft proximal region which we refer to as the vesica, and a much longer distal processus which is around two-thirds of a male’s body length (Tadler 1999). The processus is around 7.2 mm long on average in *Lygaeus equestris* (see below) and 6.8 mm long in *Lygaeus simulans* (Tadler 1999). The processus is a simple, sclerotised, hollow tube through which the ejaculate is transferred via fluid pressure at the base (Ludwig 1926). This structure is threaded along the female spermathecal duct (for visualisations, see Dougherty et al. 2015), and for insemination to be successful, it appears that the sperm have to be released at the entrance to the spermatheca at the end of this duct (Tadler 1999). The processus contains no musculature; all movement is controlled via fluid pressure from the base of the aedeagus (Ludwig 1926; Tadler 1999). The female spermathecal duct ends in a tightly coiled region that prevents the male organ from entering the spermatheca itself (Micholitsch et al. 2000; Gschwentner and Tadler 2000). The duct also possesses a valve-like structure preceding this coiled region, which may be under muscular control by the female (Gschwentner and Tadler 2000).

The *L. equestris* populations used in this experiment were derived from individuals collected from the Dolomites region of Northern Italy in 2004, and the *L. simulans* population originates from individuals collected in 2008 and 2009 from the Pratomagno region of Tuscany in Central Italy. These populations have been in continuous culture since then and go through approximately ten generations per year in the lab. Populations are maintained on organic de-husked sunflower seeds (*Helianthus anuus*) at 29 °C, with a 22:2-h light/dark cycle to prevent individuals from entering diapause. Prior to the experiments, individuals were removed from stock populations as nymphs and placed into small plastic deli tubs (108 × 82 × 55 mm). These tubs were checked every day for newly enclosed adults, which were then separated into single-sex tubs, with eight to ten individuals per tub. All tubs were provisioned with de-husked sunflower seeds ad libitum, plastic tubes containing distilled water stopped with cotton wool and a piece of dry cotton wool as shelter.

### Experiment 1: Sexual selection on processus length in *Lygaeus equestris*

The individuals used in this experiment were the same as in a previous experiment concerning sexual selection on male and female body size (see Dougherty and Shuker 2014). Briefly, mating trials were performed in which virgin males and females were allowed to freely interact for 2 h. All trials were performed using virgin, sexually mature individuals that were exactly 7 days old (post-adult eclosion). Mating trials were performed in plastic Petri dishes (55-mm diameter), at room temperature and under natural light. At the start of the trial, males and females were randomly allocated to one of four choice designs, either (1) no-choice (1 male and 1 female per dish), (2) female choice (2 males and 1 female per dish), (3) male choice (1 male and 2 females per dish) or (4) mutual choice (2 males and 2 females per dish). Note that we refer to treatment 2 as female choice because the female has a choice of mates in each dish (and vice versa for treatment 3). This does not imply though that she is able to express a choice free from interference from males during a trial. Though there is no obvious male-male aggression, multiple males may attempt to mate simultaneously with a female, and so, male-male interference may be possible (Dougherty and Shuker 2014). By allowing males and females to freely interact, we cannot eliminate potentially subtle interactions between the sexes, and so, these treatments could potentially vary the strength of both inter- and intra-sexual selection simultaneously. Individuals were marked on either the left or right side of the pronotum with a small dot of enamel paint so that they could be identified during trials. Individuals were observed continuously for 2 h in order to observe the onset of copulation. A pair was classed as being *in copula* when they were first seen in the end-to-end position typical of mating, and the entire male aedeagus was properly inserted (if the male fails to insert the aedeagus, it can easily be seen protruding from the genital capsule). After 2 h, any unmated individuals were removed (i.e. dishes in which no mating took place, but also unmated males and females from treatments 2–4). Mating pairs were then checked every 30 min for up to 6 h or until copulations ended naturally. After 6 h, any still copulating pairs were separated manually, by gently brushing them with a fine paintbrush. Pairs were only allowed to mate once to ensure an accurate measure of fertilisation success and were thus separated if a mating ended during the trial. Matings shorter than 20 min were excluded from the analysis, as previous work suggests that the minimum time required for successful insemination is slightly longer than this, at around 30 min (Tadler 1999; Micholitsch et al. 2000). These matings may end quickly because of problems with male genital deployment in the early stages of copulation. Importantly, these short matings are also not included in our measure of mating success.

Mated females were then isolated in tubs with seeds and water, and given 2 weeks to lay eggs. These tubs were checked daily for the presence of offspring. We used the successful production of offspring after 2 weeks as a proxy measure of post-copulatory selection. We hereafter refer to this measure as ‘fertilisation success’ (as we did not directly record successful sperm transfer). The production of infertile eggs was also recorded to check that females were sexually mature. Only two mated females failed to produce any eggs (including infertile ones) after 2 weeks.

After mating trials, all males were euthanised and the male genitalia were dissected out. The processus was separated from the fleshy body of the aedeagus and mounted on a microscope slide using double-sided sticky tape (following Higgins et al. 2009). These slides were then imaged using an Olympus SZX10 stereo microscope and attached camera, and measured using the image analysis program Cell^D (Soft Imaging System, Olympus Corp). Breakages may occur during dissection, and as such, only intact processes were measured. The length of the processus was measured from the middle of the ‘turning point’ to the tip (point A to B in Fig. 1), following Tadler (1999). Body length was measured for all males and for mated females. Total body length was measured on the ventral surface, from the tip of the head to the tip of the underside of the abdomen.

We assessed the repeatability of our processus length measurements by taking a second blind measurement from the image for 50 processes. Repeatability was then determined using analysis of variance (Lessells and Boag 1987).

### Experiment 2: Sexual selection on processus length in *Lygaeus simulans*

This experiment was designed to determine the strength of selection on processus length in *L. simulans* arising both before and after mating. No-choice mating trials (one male and one female per dish) were performed using virgin, sexually mature *L. simulans* individuals (between 8 and 11 days old). Trials were performed as described above. Pairs were watched continuously for 2 h, and mating attempts and copulations were recorded. After 2 h, individuals that failed to mate were euthanised. Mating pairs were checked for mating every 10 min for up to eight further hours (10 hours total), or until a copulation ended. Pairs were separated manually if they were still in copula at the end of the trial. Pairs were only allowed to mate once to ensure an accurate measure of fertilisation success and were thus separated if a mating ended during the trial. However, pairs were only separated if they were seen mating for more than 20 min (see justification above).

At the end of the mating trial, mated females were isolated in tubs and given 2 weeks to oviposit as above. If nymphs were present after 1 week, females were transferred to a new tub with fresh water. Tubs and nymphs were frozen after 2 weeks, and the number of nymphs produced by each female was counted. This gives an additional measure of post-copulatory success (offspring production) that was not measured in the previous experiment. Body length was also recorded for all males and most mated females (though some died early and so body lengths could not be measured accurately). All males were euthanised at the end of the trial, and the processus was then removed and measured as described above.

### Statistical analysis

Four measures of male reproductive success were used to quantify the strength of pre- and post-copulatory sexual selection on processus length. First, pre-copulatory sexual selection was considered, using male mating success (yes/no) as the response variable. Second, post-copulatory sexual selection was considered, using fertilisation success (presence or absence of offspring after 2 weeks) as the response variable, for males that achieved a mating. Third, overall, population-level selection was estimated using fertilisation success (yes/no) as the response variable, but this time for all males, including those that failed to mate. This measure therefore captures both male mating and subsequent fertilisation success. Finally, for experiment 2 (*L. simulans*), an additional measure of post-copulatory reproductive success was obtained by counting the number of offspring produced by mated females after 2 weeks. Importantly, both of the post-copulatory measures of reproductive success are measured in a non-competitive (single-mating, no sperm competition) context.

The strength and shape of sexual selection acting on morphological traits were tested using a regression-based approach (Lande and Arnold 1983) using generalised linear models, with mating success or fertilisation success as binary response variables or offspring number (for *L. simulans* only) as a Gaussian response variable. Processus length was included as a factor in all models. For *L. equestris* (experiment 1); experimental choice design was also included as a factor. Male and female body length and copulation duration were included as factors where appropriate (female body length was not included in models that included males that did not mate). Male body length was included as a covariate as it is correlated with processus length (see below). As copulation duration is not normally distributed and many copulations were ended manually, copulation duration was fitted as a categorical factor with two levels: either long (copulations were ended manually) or short (copulations ended naturally). Full models were fitted first, including quadratic and interaction terms where appropriate. To avoid over-parameterisation of models, any quadratic and interaction terms that were not significant were then removed in a stepwise manner.

Additionally, we estimated the strength of selection acting on male and female morphology using standardised selection differentials (Morrissey and Sakrejda 2013). We present the methods used to calculate these differentials and the results of this analysis in the online supplementary material. For both species, we calculated the strength of pre- and post-copulatory selection on male body length and processus length, and post-copulatory selection on female body length.

To visualise the shape of selection on processus length, we produced fitness surfaces using cubic splines, which are non-parametric curves that can be used to visualise complex shapes (Schluter 1988). Curves were calculated using general additive models including processus length as the single predictor variable and the smoothing parameter obtained by minimising the GCV score.

All statistical analyses were performed in R version 3.1.0 (R Development Core Team 2014).

### Post-copulatory selection in *Lygaeus simulans*: a meta-analysis

The strength of selection acting on a trait is commonly calculated using variance-standardised selection gradients (Lande and Arnold 1983; Arnold and Wade 1984). By standardising in this way, the strength of selection can be compared across multiple studies and standardised selection gradients can be seen as a measure of effect size (Kingsolver et al. 2012). If studies also present the standard error of the selection estimate, then selection can be analysed using formal meta-analyses that take sampling error into account (Morrissey and Hadfield 2011). Standardised estimates of post-copulatory selection on processus length in *L. simulans* have been published in two previous studies (Tadler 1999; Tadler et al. 1999). Though there were small methodological differences between these studies, there is nevertheless large variation in the strength of selection detected. One reason for the failure to detect selection is that some studies may have insufficient power to detect the small effect sizes typically associated with the strength of selection.

The strength of post-copulatory sexual selection on processus length in *L. simulans* across these different studies was estimated using formal meta-analysis. The variance-standardised linear or quadratic selection gradient was used as the effect size. This was first calculated using the data collected in this study following the method of Morrissey and Sakrejda (2013). Five pairs (linear and quadratic) of effect size estimates were obtained: one from this experiment, two derived from previous published studies (Tadler 1999; Tadler et al. 1999) and two re-calculated using data from a recently published experiment (Dougherty et al. 2015). See the online supplementary material for details on the methods used to calculate selection gradients and the final effect sizes used in the analysis (Table S8). Selection gradients were calculated using univariate regression models, i.e. models do not include covariates (but they do include both linear and quadratic terms). These gradients describe the total selection acting on a trait, including indirect selection arising through selection on correlated traits (these may be more correctly referred to as selection differentials: Kingsolver et al. 2012).

Analyses were performed separately for the linear and quadratic selection gradients. The variance associated with each effect size was calculated as SE^2^. For both datasets, the mean effect size was estimated using a random-effects meta-analysis. The mean effect size estimate was considered to be significantly different from zero if the 95 % confidence intervals did not include zero. The *I*^2^ statistic was used to determine the amount of heterogeneity in effect sizes across studies; this gives the percentage of variation in effect sizes due to heterogeneity rather than by chance (Higgins et al. 2003). Finally, a mixed-effects meta-analytic model (random-effects model with a categorical fixed factor: Nakagawa and Santos 2012) was used to test if the mean effect size for each type of selection differed due to study author, and a meta-regression model was used to test if effect size was significantly affected by study year. This analysis was performed in R 3.0.1 (R Development Core Team 2014) using the Metafor package (Viechtbauer 2010).

## Results

### Experiment 1: Sexual selection on processus length in *Lygaeus equestris*

Processus measurements were obtained for 174 males in total (*N* = 39, 54, 26 and 55 individuals from treatments 1, 2, 3 and 4, respectively), of which 64 mated (*N* = 15, 17, 9 and 23 individuals from treatments 1, 2, 3 and 4, respectively). Repeatability of processus measurement was very high (*r* = 0.98). Average processus length was 7.22 mm (s.d. = 0.17 mm), which is over two-thirds the total body length of males (mean = 10.22 mm, s.d. = 0.32 mm). Average female body length was 11.25 mm (s.d. = 0.38 mm, *N* = 64). Processus length was significantly correlated with male body length (*r*_172_ = 0.4, *P* < 0.001).

**Fig. 2:**
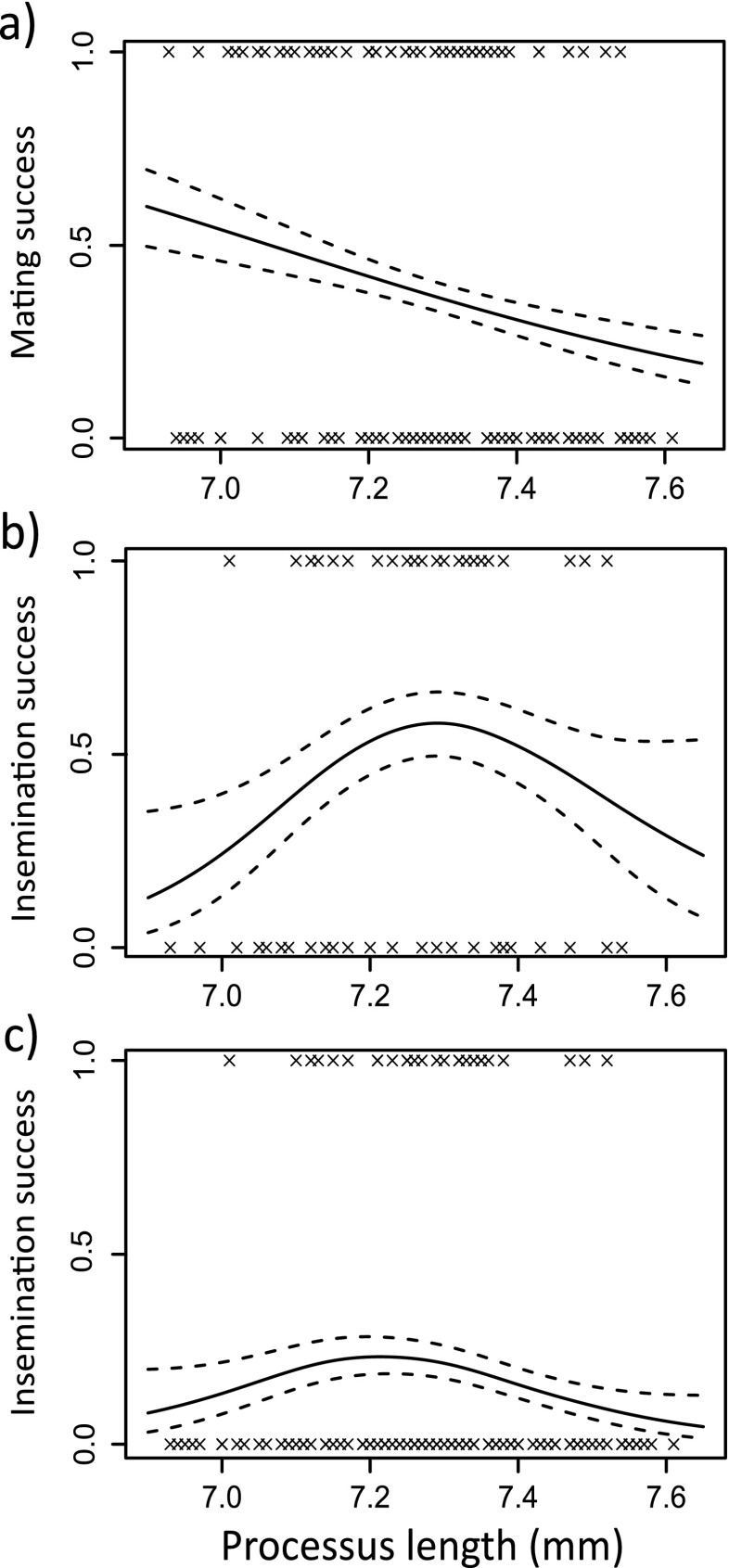
Fitness functions showing selection on male processus length in *L. equestris* for three measures of male reproductive success: **a** male mating success (mated or non-mated, *N* = 174), **b** male insemination success (production of offspring) for mated males (*N* = 64) and **c** male insemination success (production of offspring) for all males, including those that did not mate (*N* = 174). *Dashed lines* indicate 1 standard error above and below the predicted line

**Fig. 3:**
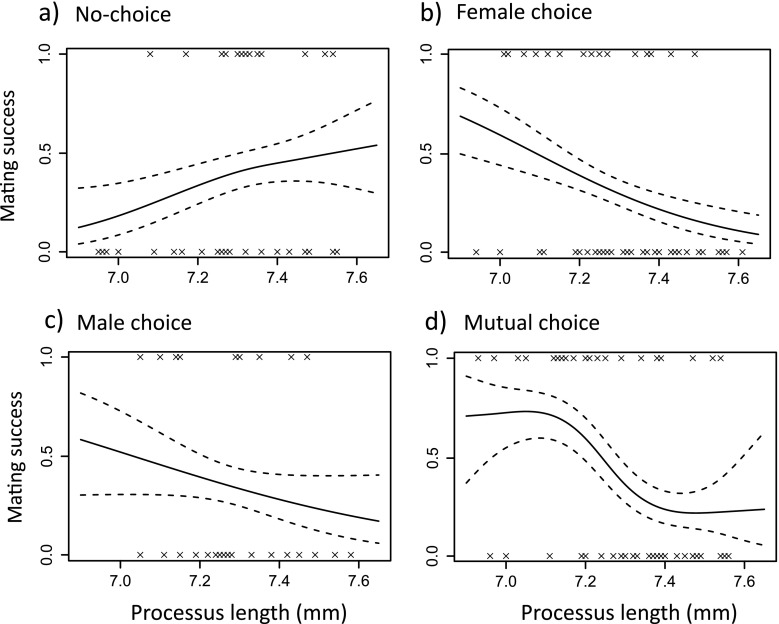
Fitness functions showing pre-copulatory selection on male processus length in *L. equestris* for the four experimental choice designs: **a** no-choice (*N* = 39), **b** female choice (*N* = 54), **c** male choice (*N* = 26) and **d** mutual choice (*N* = 55). *Dashed lines* indicate 1 standard error above and below the predicted line

We first consider pre-copulatory selection on male processus length arising from differential mating success. Across all choice designs, male mating success was not associated with processus length (binary logistic GLM; linear: *χ*^2^_1_ = 1.39, *P* = 0.24, quadratic: *χ*^2^_1_ = 0.56, *P* = 0.45; Fig. 2a). However, male mating success was significantly influenced by the interaction between linear processus length and choice design (*χ*^2^_1_ = 6.1, *P* = 0.014), so that significant negative pre-copulatory selection on processus length was only seen in mating trials in which two females were present (Fig. 3). Male mating success was significantly lower under a female-biased choice design treatment than under the other three designs (*χ*^2^_1_ = 6.15, *P* = 0.013); however, this is probably driven by the low sample size of that treatment (only nine matings were observed). Male mating success was not significantly associated with male body length (linear: *χ*^2^_1_ = 0.055, *P* = 0.82, quadratic: *χ*^2^_1_ = 0.44, *P* = 0.51).

In terms of post-copulatory selection on processus length arising from differential fertilisation success, 30 out of 64 mated females laid fertile eggs. For those males that mated, processes of intermediate length were significantly more likely to lead to the production of offspring (quadratic: *χ*^2^_1_ = 7.84, *P* = 0.005; Fig. 2b). Selection analysis indicated a quadratic selection gradient of −0.41 (*SE* = 0.098; Table S1). Post-copulatory selection on processus length did not vary according to choice design (interaction between quadratic processus length and choice design; *χ*^2^_1_ = 1.52, *P* = 0.22). Fertilisation success was also significantly lower for males of intermediate body length (quadratic: *χ*^2^_1_ = 10.92, *P* = 0.001). Selection analysis indicated a quadratic (disruptive) selection gradient on body length of 0.37 (SE = 0.16; Table S3). Fertilisation success was not significantly influenced by female body length (linear: *χ*^2^_1_ = 3.26, *P* = 0.071, quadratic: *χ*^2^_1_ = 3.2, *P* = 0.073) or by choice design (*χ*^2^_1_ = 1.48, *P* = 0.22). Finally, longer copulations were also significantly more likely to result in successful fertilisation (*χ*^2^_1_ = 5.68, *P* = 0.02), with only a single copulation shorter than 200 min resulting in offspring. Twenty-six out of 64 copulations were ended manually. Males did not have to copulate for as long in order to successfully fertilise small females compared to large females (interaction between female body length and copulation duration, *χ*^2^_1_ = 6.08, *P* = 0.014). There was no significant correlation between processus length and copulation duration for mated males (*r*_s_ = −0.2, *N* = 64, *P* = 0.11).

If we consider ‘overall’ selection (fertilisation success for all males), again males with an intermediate processus length were significantly more likely to produce a successful fertilisation (quadratic: *χ*^2^_1_ = 7.35, *P* = 0.007; Fig. 2c). Selection analysis indicated a quadratic selection gradient of −0.38 (SE = 0.11; Table S1). Overall fertilisation success was significantly lower when tested using a female-biased choice design (*χ*^2^_1_ = 4.3, *P* = 0.038), which again is probably driven by the low sample size of this treatment. The association between overall fertilisation success and processus length varied according to choice design (interaction between processus length and choice design; *χ*^2^_1_ = 4.21, *P* = 0.04) with stabilising selection seen only in the two choice designs with an equal sex ratio; however, the number of successful fertilisations in each treatment is low (Fig. S1). Overall fertilisation success was not associated with male body length (linear: *χ*^2^_1_ = 0.19, *P* = 0.67, quadratic: *χ*^2^_1_ = 1.74, *P* = 0.19).

### Experiment 2: Sexual selection on processus length in *Lygaeus simulans*

Mating trials were performed using 140 pairs, of which 102 mated. Males had an average processus length of 6.76 mm (s.d. = 0.19 mm). One male had a very short processus (5.61 mm with tip still intact), though removal of this outlier did not change the mean greatly (*N* = 139, mean = 6.77 mm, s.d. = 0.16 mm). This male was included in the analysis but was removed when plotting cubic splines, as outliers can have a strong effect on curve fitting. As the male was unmated, it was only included in the analysis considering male mating success; nevertheless, removing this outlier had no effect on the results. Average male body length was 10.19 mm (s.d. = 0.36 mm), and average female body length was 11.44 mm (s.d. = 0.39 mm, *N* = 86). There was a significant correlation between male body length and processus length (*r*_138_ = 0.46, *P* < 0.001).

**Fig. 4:**
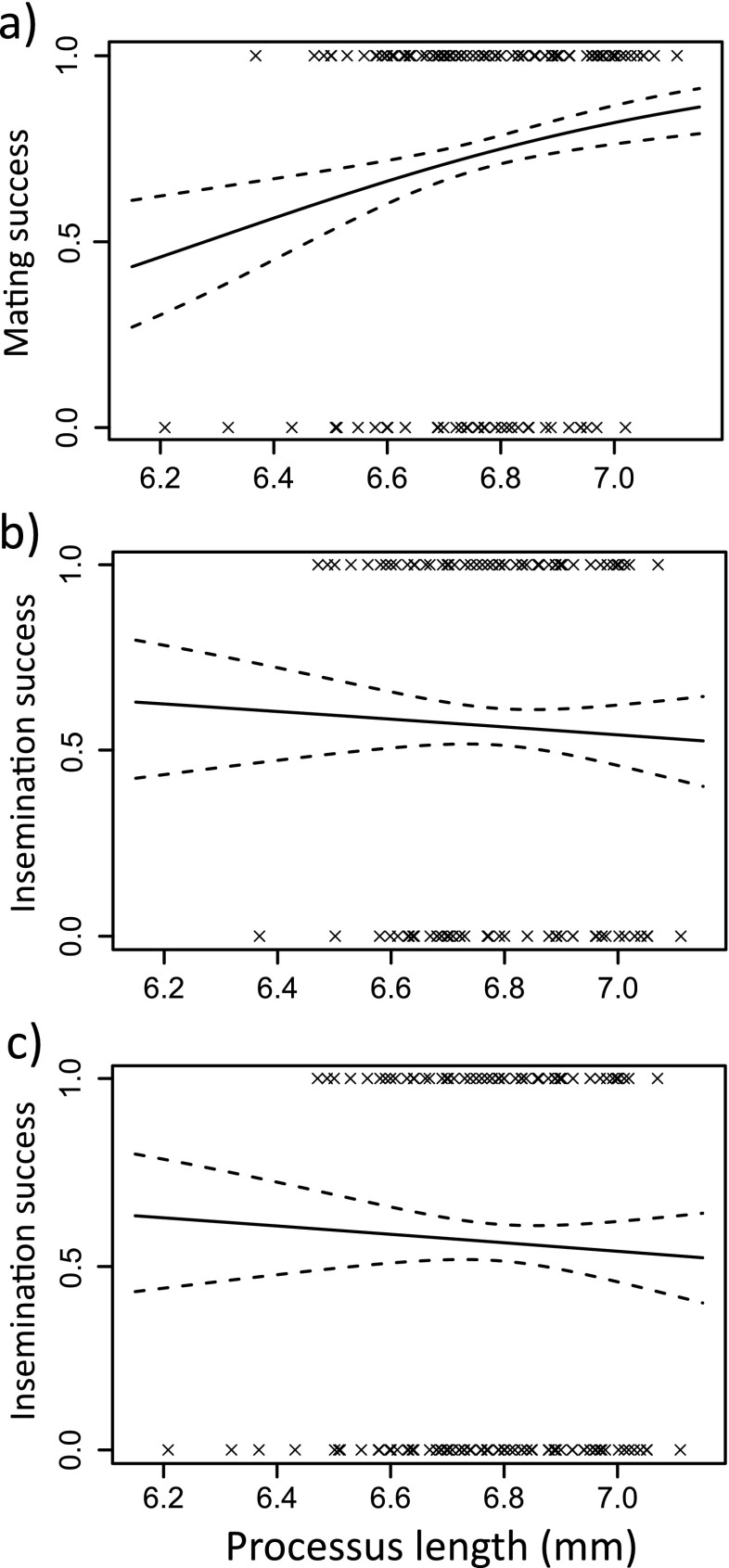
Fitness function showing **a** pre-copulatory selection on male processus length for all males (*N* = 140), **b** post-copulatory selection on male processus length for mated males only (*N* = 101) and **c** post-copulatory selection on male processus length for all males (*N* = 140), in *L. simulans. Dashed lines* indicate 1 standard error above and below the predicted line

We first consider pre-copulatory selection on morphology arising from differential mating success. Male mating success was not significantly associated with processus length (linear: *χ*^2^_1_ = 0.31, *P* = 0.58, quadratic: *χ*^2^_1_ = 0.0001, *P* = 0.99; Fig. 4a). However, larger males were more likely to achieve a mating (linear: *χ*^2^_1_ = 5.9, *P* = 0.015, quadratic: *χ*^2^_1_ = 0.22, *P* = 0.64). Selection analysis indicated a linear selection gradient on body length of 0.14 (SE = 0.05; Table S6).

We next consider post-copulatory selection arising via male fertilisation success for mated pairs. Fertility data was obtained for 101 mated females and body length measurements for 86 of these. Twenty-five of 102 matings were ended manually. The likelihood of fertilisation increased with the time spent in copula (*χ*^2^_1_ = 36.02, *P* < 0.001). Contrary to previous studies, fertilisation success was not associated with quadratic processus length (*χ*^2^_1_ = 0.08, *P* = 0.77; Fig. 4b) nor was there a significant linear effect (*χ*^2^_1_ = 1.48, *P* = 0.22). Fertilisation success was also not associated with male body length (linear: *χ*^2^_1_ = 0.14, *P* = 0.71, quadratic: *χ*^2^_1_ = 1.86, *P* = 0.17). However, larger females were significantly more likely to be successfully fertilised (linear: *χ*^2^_1_ = 10.64, *P* = 0.001, quadratic: *χ*^2^_1_ = 1.56, *P* = 0.21). Selection analysis indicated a linear selection gradient of 0.33 (SE = 0.09; Table S7). This is likely driven by the positive correlation between female body length and copulation duration (Spearman’s rank correlation, *N* = 86, *r*_*s*_ = 0.23, *P* = 0.032).

When considering overall fertilisation success (including males that did not mate), the production of offspring was more likely when pairs mated for longer (*χ*^2^_1_ = 37.73, *P* < 0.001) but was not associated with male body length (linear: *χ*^2^_1_ = 0.08, *P* = 0.78, quadratic: *χ*^2^_1_ = 0.001, *P* = 0.97) or processus length (linear: *χ*^2^_1_ = 1.41, *P* = 0.24, quadratic: *χ*^2^_1_ = 0.24, *P* = 0.63; Fig. 4c).

Finally, we consider post-copulatory sexual selection arising through differential offspring production, in those pairs that successfully produced offspring. Larger females produced significantly more offspring (linear: *F*_1, 43_ = 8.61, *P* = 0.005, quadratic: *F*_1, 40_ = 0.03, *P* = 0.87). However, offspring number was not significantly influenced by copulation duration (*F*_1, 43_ = 0.48, *P* = 0.49), male body length (linear: *F*_1, 43_ = 3.07, *P* = 0.09, quadratic: *F*_1, 40_ = 0.81, *P* = 0.37) or processus length (linear: *F*_1, 43_ = 0.01, *P* = 0.91, quadratic: *F*_1, 40_ = 0.005, *P* = 0.95).

### Meta-analysis

There was no significant linear post-copulatory selection on processus length across the five effect sizes obtained (mean = 0.03, 95 % CI lower = −0.06, 95 % CI upper = 0.12). The amount of heterogeneity across effect sizes was too small to calculate *I*^2^. There was no significant effect of author (*Q*_*M* 1_ = 0.61, *P* = 0.44) or study year (*Q*_*M* 1_ = 0.66, *P* = 0.42) on the strength of linear selection.

**Fig. 5:**
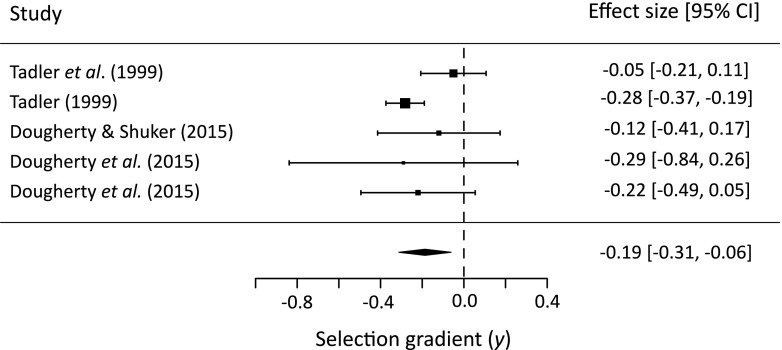
Forest plot showing the quadratic selection gradient (*γ*) and associated 95 % CI of the effect sizes included in the meta-analysis. The sizes of the squares represent the relative weightings of each effect size in the model. The mean effect size estimate produced using a random-effects model is represented by the centre of the diamond, with the width of the diamond representing the 95 % CI of the estimate

Overall, the quadratic selection gradient was significantly negative, indicating significant stabilising selection on processus length across all studies (mean = −0.19, 95 % CI lower = −0.31, 95 % CI upper = −0.06; Fig. 5). The percentage heterogeneity (*I*^2^) was 44 % (this is suggested to be ‘moderate’: Higgins et al. 2003). There was no significant effect of author (*Q*_*M* 1_ = 0.004, *P* = 0.95) or study year (*Q*_*M* 1_ = 0.003, *P* = 0.96) on the strength of quadratic selection.

## Discussion

We have shown that the size of the male intromittent organ in *L. equestris* and *L. simulans* is subject to contrasting episodes of selection acting at different stages of mating. Prior to mating, there is negative, linear selection on processus length in *L. equestris*, but only in mating trials in which two males were present. Therefore, the social environment has an influence on the shape of pre-copulatory selection on male intromittent organ length in *L. equestris*. Additionally, during mating, there is significant stabilising selection on processus length in both species. However, such selection was only apparent in *L. simulans* when combining multiple estimates of selection across studies, with selection varying in strength among those studies. The net outcome of both pre- and post-copulatory episodes of selection is significant stabilising selection on processus length in both species. When significant selection was detected, the selection gradients were reasonably large (between absolute values of 0.14 and 0.41), at least in comparison to selection estimates from wild populations (Kingsolver et al. 2001, 2012).

### Pre-copulatory selection on processus length

We detected significant negative pre-copulatory selection on processus length in *L. equestris*, but only for males that were used in mating trials for which rivals were present. This suggests that the social environment strongly influences the nature of pre-copulatory selection on processus length. This is confirmed by the fact that pre-copulatory selection on processus length was not seen in the *L. simulans* experiment, for which only a ‘no-choice’ mating design was used. Importantly, significant pre-copulatory selection on processus length was detected in *L. equestris* despite the fact that the processus is stored inside the male genital capsule before mating. There is thus no way in which the processus is able to interact with, or be assessed by, the female until intromission has been achieved. Furthermore, we have shown previously that both the experimental reduction of processus length and damage to the processus inflicted naturally in our laboratory population have no effect on male mating success in *L. simulans* (Dougherty et al. 2015; Dougherty and Shuker, 2015b). Instead, the most likely explanation for this pattern is that selection is arising indirectly, due to selection on a correlated trait. We can rule out male body length as the cause of this indirect selection, as the analysis controls for this. One possibility is that processus length is strongly correlated with another male genital trait. For example, the male genital claspers are used to open the female ovipositor prior to mating and could potentially be under strong selection as males need to overcome female resistance in this species (Sillén-Tullberg 1981; Shuker et al. 2006). This indirect selection could explain why selection on processus length was only seen in the choice designs in which two males were present. Male-male competition is predicted to be stronger in these contexts, so that male traits that are important during such competition will come into play. For example, clasper size or shape may be important for deciding the winner of mating scrambles. However, the strength and direction of pre-copulatory selection on clasper morphology have not been assessed in this species. An alternative explanation is that pre-copulatory selection arises via female choice, as mate choice tends to be stronger when two options are available compared to one (Dougherty and Shuker 2015a). Again though, male processus length cannot be assessed directly by the female prior to mating, and so there would have to be indirect selection arising from a correlated trait that we have not measured.

Nevertheless, this putative indirect pre-copulatory selection will still able to influence genital evolution if it is consistent, especially if genital traits are strongly correlated. Pre-copulatory selection has been shown to act in other species on external genital traits (e.g. Kahn et al. 2009; Grieshop and Polak 2012) and less commonly on internal genital traits (Simmons et al. 2009; Xu and Wang 2010). Pre-copulatory selection on genital morphology might be expected for traits involved in maintaining genital contact during mating, and these traits may remain outside the female or be associated with the intromittent organ (e.g. Simmons et al. 2009). However, for traits such as the processus, which is clearly not involved in maintaining genital contact, such selection is unexpected. We suggest that where possible, pre-copulatory selection should be tested as a mechanism of genital evolution, if only to rule out its effect. This should not require too much of a change in experimental design: Researchers are already essentially recording male mating in any study of post-copulatory selection in which some males fail to mate, but these males are typically discarded before genitalia are measured.

Importantly, the strength of pre-copulatory selection on processus length varies significantly according to social context in *L. equestris*. Until this pre-copulatory selection on processus length is explained, it is hard to interpret why we have detected an effect of social context on processus length but not on other morphological traits (Dougherty and Shuker 2014 found no influence of the social environment on pre-copulatory selection on male or female body length). However, one important implication from this result is that the strength of pre-copulatory selection on processus length in the wild will thus depend on how often *L. equestris* males encounter mates in isolation or in the presence of rivals. If many rivals are present during the breeding season (e.g. see Solbreck 1972) then pre-copulatory selection on processus length may be negative for a significant proportion of the population. Confirming the effect of social environment on *L. simulans* will be an interesting corollary to the current study.

We cannot rule out the possibility that we may have failed to detect pre- or post-copulatory significant selection on certain traits due to limited statistical power. We have addressed this problem directly when considering post-copulatory selection in *L. simulans* by using meta-analysis to combine multiple estimates of selection found here and in previous studies (see below). We were unable to do this for pre-copulatory selection on processus length in *L. simulans*; however, we do not think that our failure to detect significant selection in this case is solely due to a lack of power. This is because we were able to detect significant linear pre-copulatory selection on processus length in *L. equestris* with relatively small sample sizes of 54 and 55 (when considering experimental choice treatments individually: Table S2). The sample size for the *L. simulans* experiment was almost three times as large as this (*N* = 140). From this, we can infer that the strength of pre-copulatory selection on processus length in *L. simulans* is much weaker than for *L. equestris*, and this difference is unlikely to be an artefact of small sample sizes. We suggest that the only relevant difference between these two experiments was the social environment.

### Post-copulatory selection on processus length

Overall, we detected significant stabilising post-copulatory selection on processus length in both *L. equestris* and *L. simulans*. Though we were unable to detect stabilising post-copulatory selection on processus length in our *L. simulans* experiment reported here, significant stabilising selection was detected after combining multiple estimates from this and other published studies using meta-analysis. This is likely because most of the studies in the analysis (including this one) had limited power on their own to detect a significant effect, given that the quadratic selection gradient (representing stabilising post-copulatory selection on processus length) for *L. simulans* is weaker than for *L. equestris* (*γ* = −0.19 and −0.41, respectively). The forest plot (Fig. 5) shows that there is a large difference in the variance associated with each effect size for studies considering *L. simulans*. The variance in this case is derived from the reported standard error of each selection estimate and so reflects both the sample size of the experiment and also the variability in success across male phenotypes. One reason why the Tadler (1999) study especially has a very low variance compared to the other studies might be that related females were deliberately used to reduce the variation in cryptic female choice. Nevertheless, one of the strengths of meta-analysis is its ability to combine several effect size estimates of low power. Moving beyond fertilisation success in *L. simulans*, in our experiment, there was no relationship between processus length and the number of offspring produced following a fertile mating.

The processus in both species is therefore under significant stabilising selection despite its great length. Why the processus has evolved to be so long in the first place is still unclear, though the extreme length would suggest that sexual selection has played a role (Simmons, 2014). Genital traits are commonly shown to be under stabilising selection (e.g. Tadler 1999; Simmons et al. 2009; Simmons 2014), and this has been predicted to arise via several mechanisms, including female choice to fit the average genital tract, or natural selection to prevent inter-species mating (House et al. 2013; Simmons 2014). However, we recently used experimental ablation to confirm that processus length directly influences male post-copulatory reproductive success in *L. simulans* (Dougherty et al. 2015), strongly supporting a role for sexual selection in maintaining the length of the processus in this species.

Why do males with processes either significantly longer or shorter than the population average have reduced fertilisation success? Such a relationship would make intuitive sense if the length of the female reproductive tract was similar to that of the male processus, but that is not the case. Instead, the female spermathecal duct is significantly shorter than the processus (around 1.9 mm long in *L. simulans*: Gschwentner and Tadler 2000), so that a large proportion of the processus remains in the bursa during mating (Dougherty et al. 2015). For some reason then, males with short processes cannot simply thread more of the processus into the spermathecal duct, despite appearing to have plenty to spare. We suggest two other mechanisms in which processus length could be important for fertilisation success. The first is that the length of the processus may be important in a structural sense, for example if it makes the structure more flexible, or if the number of coils made within the bursa is important for positioning the tip at the entrance to the spermathecal duct (Dougherty et al. 2015). Alternatively, this result, coupled with the presence of a valve at the entrance to the spermatheca, could be explained as the result cryptic female choice, with females actively preventing unwanted males from achieving insemination (Eberhard 1996; Gschwentner and Tadler 2000).

In both *L. equestris* and *L. simulans*, matings frequently fail to lead to the production of offspring (Tadler 1999; Dougherty and Shuker 2014; Greenway and Shuker 2015). We assume in these experiments that failure to produce offspring following mating reflects a failure to inseminate a female. However, this may not be true; instead, sperm may be successfully deposited in the spermatheca but not utilised by the female. For example, Tadler et al. (1999) found that in 12 of 67 matings (18 %), sperm was present in the spermathecae, but no fertile eggs were produced after 45 days. It is unclear why these sperm were not used to fertilise the female’s eggs. The proxy measure of insemination success used here therefore slightly underestimates the likelihood of a male’s sperm reaching the spermatheca. We also note that post-copulatory sexual selection was tested using a single-mating design, which means that there is no direct competition between rival male ejaculates. Though the exact level of polyandry in wild populations of both species has not been investigated, females do appear to mate multiple times between oviposition events in some cases (Solbreck 1972; Sillén-Tullberg 1981). Therefore, the strength and shape of post-copulatory sexual selection on processus length in the wild may be different to that seen here.

### Combining selection

Sexual selection may act both before, during or after copulation. However, historically, these different episodes of selection have been considered in isolation (Hunt et al. 2009; Kvarnemo and Simmons 2013). Yet, if we want to understand how selection acts on populations in the wild, then we need to estimate total selection arising from multiple episodes, for example by combining intrasexual and intersexual selection (Hunt et al. 2009), or pre- and post-copulatory selection (Young et al. 2010; Pélissié et al. 2014). Importantly, individual behavioural or morphological traits may be subject to contrasting episodes of selection (Bonduriansky and Rowe 2003). For example, in the water strider *Gerris lacustris*, pre-copulatory selection favours large males, whereas post-copulatory selection favours small males (Danielsson 2001). Despite this, studies looking at multiple episodes of selection acting on genital traits are rare (but see Gasparini et al. 2011; House et al. 2013; LeVasseur‐Viens et al. 2015; Frazee and Masly 2015). This is surprising given that genital traits may have complex functions that can influence both pre- and post-copulatory reproductive success. For example, external grasping structures could potentially function both to initiate copulation and to extend duration so that sperm transfer can take place (Eberhard 1985; Simmons 2014).

We combined pre- and post-copulatory measures of selection by quantifying the strength of selection arising through male fertilisation success for all males, including those who failed to mate. In *L. simulans* combining selection in this way led to no changes in the shape of selection on processus length, which is unsurprising as no significant pre- or post-copulatory selection was detected. In *L. equestris*, this combined measure indicated strongly stabilising selection on male processus length. This suggests that the linear pre-copulatory selection on processus length seen in *L. equestris* is insufficient to overcome (or in part contributes to) the strong stabilising post-copulatory selection in the population. This method of combining both measures of selection requires that morphological data are also measured for individuals that do not mate during mating trials, which will of course require more data collection. This approach could also be extended to more natural competitive situations if methods for identifying offspring, such as genotyping or sterile male techniques, are available. This is analogous to recent studies that attempt to estimate the overall contributions of pre- and post-copulatory reproductive success to total fitness, albeit without correlating this to specific phenotypic traits (e.g. Pélissié et al. 2014).

## Electronic supplementary material

ESM 1 (DOCX 162 kb)
