# Supplementary material for: Variation in pre- and post-copulatory sexual selection on male genital size in two species of lygaeid bug
Source: Behav Ecol Sociobiol. 2016 Mar 2;70:625–37. doi: 10.1007/s00265-016-2082-6 (PMC4788681; doi:10.1007/s00265-016-2082-6)
Supplement: Supplementary file 1 — (DOCX 162 kb) [file 265_2016_2082_MOESM1_ESM.docx]

# Supplementary material

## Part 1: selection analysis

**Obtaining selection differentials**

We estimated the strength of sexual selection acting on male and female morphological traits using standardized selection differentials (Morrissey and Sakrejda 2013). The selection gradient is a common metric that represents the relationship between relative fitness and variation in a quantitative trait measured in standard deviation units, and is standardized by setting the trait variance to 1 prior to measurement (Kingsolver et al. 2001). The selection differential includes both the strength of direct selection acting on the trait, and also the strength of indirect selection due to correlated traits (Kingsolver et al. 2012). The difference comes from whether the statistical model used to estimate the strength of selection includes covariates or not: if there is no attempt to control for covariates (so that only one explanatory factor is included in the model), then some of the selection measured may arise from selection on correlated traits, and so this is a selection differential. Note that this is more appropriate if the aim is to record the strength of overall selection acting on a given trait.

Standardised selection differentials were calculated using the R package GSG (Morrissey and Sakrejda 2013). The GSG package allows selection gradients and differentials to be calculated from non-parametric general additive models, and allows the calculation of standard errors and *P* values associated with these estimates via bootstrapping. For all models the morphological trait in question (processus length, male body length or female body length) was the only explanatory factor. The package calculates the strength of linear (β) and quadratic (γ; stabilising or disruptive) selection. Note that stabilising selection on a trait results in a negative value of quadratic selection, whereas disruptive selection results in a positive value of quadratic selection (Mitchell-Olds and Shaw 1987). Selection differentials were calculated using three estimates of reproductive fitness: pre-copulatory success (whether an individual mated or not), post-copulatory success (whether a mating led to the production of offspring), and a combination of the two (whether an individual produced fertile offspring, including mated and non-mated individuals).

The strength of selection on male and female morphology was first considered for *L. equestris*. The strength of selection on male processus length across all four experimental choice treatments is presented in **Table S1**, calculated separately for the three measures of reproductive fitness. The strength of pre-copulatory selection on male processus length in each of the four experimental choice treatments is presented in **Table S2**. The strength of selection on male body length across all four experimental choice treatments is presented in **Table S3**, calculated separately for the three measures of reproductive fitness. Finally, the strength of post-copulatory selection on female body length is presented in **Table S4**.

Next the strength of selection on male and female morphology was considered for *L. simulans*. The strength of selection on male processus length is presented in **Table S5**, calculated separately for the three measures of reproductive fitness. The strength of selection on male body length is presented in **Table S6**, calculated separately for the three measures of reproductive fitness. Finally, the strength of post-copulatory selection on female body length is presented in **Table S7**.

**References**

Kingsolver JG, Diamond SE, Siepielski AM, Carlson SM (2012) Synthetic analyses of phenotypic selection in natural populations: lessons, limitations and future directions. Evolutionary Ecology 26:1101–1118.

Kingsolver JG, Hoekstra HE, Hoekstra JM, Berrigan D, Vignieri SN, Hill CE, Hoang A, Gilbert P, Beerli P (2001) The strength of phenotypic selection in natural populations. The American Naturalist 157:245–261.

Mitchell-Olds T, Shaw RG (1987) Regression analysis of natural selection: statistical inference and biological interpretation. Evolution 41:1149–1161.

Morrissey MB, Sakrejda K (2013) Unification of regression-based methods for the analysis of natural selection. Evolution 67:2094–2100.

**Supplementary tables: *Lygaeus equestris***

**Table S1.** The strength of linear and quadratic selection on male processus length in *L. equestris*, for the three measures of reproductive success.

| Selection | *N* | Term | Estimate | *SE* | *P* |
| --- | --- | --- | --- | --- | --- |
| Pre-copulatory | 174 | Linear (*β*) | -0.25 | 0.088 | <0.001 |
|  |  | Quadratic (*γ*) | -0.093 | 0.12 | 0.51 |
| Post-copulatory | 64 | Linear (*β*) | 0.086 | 0.1 | 0.33 |
|  |  | Quadratic (*γ*) | -0.41 | 0.098 | 0.012 |
| Combined | 174 | Linear (*β*) | -0.19 | 0.11 | 0.09 |
|  |  | Quadratic (*γ*) | -0.38 | 0.11 | 0.006 |

**Table S2**. The strength of linear and quadratic pre-copulatory selection on male processus length in *L. equestris*, split by choice design.

| Treatment | *N* | Term | Estimate | *SE* | *P* |
| --- | --- | --- | --- | --- | --- |
| 1 | 39 | Linear | 0.126 | 0.216 | 0.574 |
|  |  | Quadratic | -0.352 | 0.295 | 0.184 |
| 2 | 54 | Linear | -0.414 | 0.163 | 0.022 |
|  |  | Quadratic | -0.065 | 0.225 | 0.714 |
| 3 | 26 | Linear | -0.246 | 0.267 | 0.394 |
|  |  | Quadratic | 0.051 | 0.466 | 0.988 |
| 4 | 55 | Linear | -0.424 | 0.133 | 0.006 |
|  |  | Quadratic | 0.050 | 0.202 | 0.622 |

**Table S3**. The strength of linear and quadratic selection on male body length in *L. equestris*, for the three measures of reproductive success.

| Selection | *N* | Term | Estimate | *SE* | *P* |
| --- | --- | --- | --- | --- | --- |
| Pre-copulatory | 174 | Linear (*β*) | -0.09 | 0.1 | 0.35 |
|  |  | Quadratic (*γ*) | -0.12 | 0.11 | 0.31 |
| Post-copulatory | 64 | Linear (*β*) | 0.08 | 0.12 | 0.51 |
|  |  | Quadratic (*γ*) | 0.37 | 0.16 | 0.04 |
| Combined | 174 | Linear (*β*) | 0.07 | 0.19 | 0.71 |
|  |  | Quadratic (*γ*) | 0.16 | 0.25 | 0.52 |

**Table S4**. The strength of linear and quadratic post-copulatory selection on female body length in *L. equestris*.

| Selection | *N* | Term | Estimate | *SE* | *P* |
| --- | --- | --- | --- | --- | --- |
| Post-copulatory | 174 | Linear (*β*) | 0.07 | 0.14 | 0.63 |
|  |  | Quadratic (*γ*) | 0.01 | 0.19 | 0.95 |

**Supplementary tables: *Lygaeus equestris***

**Table S5**. The strength of linear and quadratic selection on male processus length in *L. simulans*, for the three measures of reproductive success.

| Selection | *N* | Term | Estimate | *SE* | *P* |
| --- | --- | --- | --- | --- | --- |
| Pre-copulatory | 139 | Linear (*β*) | 0.087 | 0.05 | 0.064 |
|  |  | Quadratic (*γ*) | -0.03 | 0.066 | 0.582 |
| Post-copulatory | 101 | Linear (*β*) | -0.041 | 0.088 | 0.674 |
|  |  | Quadratic (*γ*) | -0.12 | 0.147 | 0.436 |
| Combined | 139 | Linear (*β*) | -0.043 | 0.089 | 0.644 |
|  |  | Quadratic (*γ*) | -0.11 | 0.142 | 0.47 |

**Table S6**. The strength of linear and quadratic selection on male body length in *L. simulans*, for the three measures of reproductive success.

| Selection | *N* | Term | Estimate | *SE* | *P* |
| --- | --- | --- | --- | --- | --- |
| Pre-copulatory | 139 | Linear (*β*) | 0.14 | 0.053 | 0.01 |
|  |  | Quadratic (*γ*) | -0.05 | 0.056 | 0.21 |
| Post-copulatory | 102 | Linear (*β*) | -0.02 | 0.091 | 0.83 |
|  |  | Quadratic (*γ*) | -0.03 | 0.12 | 0.86 |
| Combined | 139 | Linear (*β*) | 0.12 | 0.11 | 0.27 |
|  |  | Quadratic (*γ*) | -0.09 | 0.13 | 0.42 |

**Table S7.** The strength of linear and quadratic post-copulatory selection on female body length in *L. simulans*.

| Selection | *N* | Term | Estimate | *SE* | *P* |
| --- | --- | --- | --- | --- | --- |
| Post-copulatory | 102 | Linear (*β*) | 0.33 | 0.09 | <0.001 |
|  |  | Quadratic (*γ*) | 0.08 | 0.12 | 0.46 |

## Part 2: Effect sizes for meta-analysis

We performed two meta-analyses to estimate the strength of post-copulatory sexual selection on processus length in *Lygaeus simulans*. To do this standardized linear or quadratic selection differentials were used as the effect size measures. Five pairs of effect sizes were obtained: one calculated using the data collected for this study (**Table S5**), two calculated using the data collected for a recently published study by the authors (Dougherty et al. 2015) and two from results presented in two previous published studies (Tadler 1999; Tadler et al. 1999). Full information regarding the effect sizes used in the analysis can be seen in **Table S8**. For the present study and Dougherty et al. (2015) the raw data was available, and the strength of selection was calculated as described above. For Dougherty et al. (2015), data was available from two separate experiments, and so two effect sizes were obtained. Only those males that did not have the processus length manipulated (‘sham males’) were used to estimate selection. For Tadler et al. (1999) and Tadler (1999) the selection differentials and associated standard errors were taken from presented values.

All effect sizes considered the strength of post-copulatory selection in mated males; however fitness was recorded in different ways. For the present experiment and Dougherty et al. (2015), post-copulatory selection was calculated using the successful production of offspring (as a binary trait) as the measure of fitness. Tadler (1999) used the actual presence of sperm in the spermatheca as the measure of insemination success. Finally, Tadler et al. (1999) present selection found from the regression of processus length against the number of offspring produced after a single mating (selection gradients arising through insemination success are presented but without standard errors).

**Table S8**. Standardised selection gradients showing the strength of post-copulatory selection on processus length in *L. simulans*. We used meta-analysis to determine the overall strength of linear and quadratic selection across the studies. The selection gradients (*β* and *γ*) were used as measures of effect size along with the associated variance (var), calculated as SE^2^. *N*= the number of mated females used to assess selection.

|  |  |  | Linear selection | | | | Quadratic selection | | | |
| --- | --- | --- | --- | --- | --- | --- | --- | --- | --- | --- |
| Study | **N** | **Response** | ***β*** | **SE (β)** | **var (β)** | ***P* (β)** | ***γ*** | **SE (γ)** | **var (γ)** | ***P* (γ)** |
| Tadler et al. 1999 | 67 | Fecundity | 0.03 | 0.15 | 0.023 | 0.86 | -0.05 | 0.08 | 0.006 | 0.58 |
| Tadler 1999 | 102 | Insemination | 0.079 | 0.077 | 0.006 | 0.3 | -0.282 | 0.047 | 0.002 | 0.002 |
| This study | 102 | Fertilisation | -0.041 | 0.088 | 0.008 | 0.67 | -0.12 | 0.15 | 0.023 | 0.436 |
| Dougherty et al. 2015 | 31 | Fertilisation | 0.18 | 0.19 | 0.036 | 0.47 | -0.29 | 0.28 | 0.078 | 0.16 |
| Dougherty et al. 2015 | 50 | Fertilisation | -0.01 | 0.11 | 0.012 | 0.83 | -0.22 | 0.14 | 0.02 | 0.15 |

## Supplementary figures


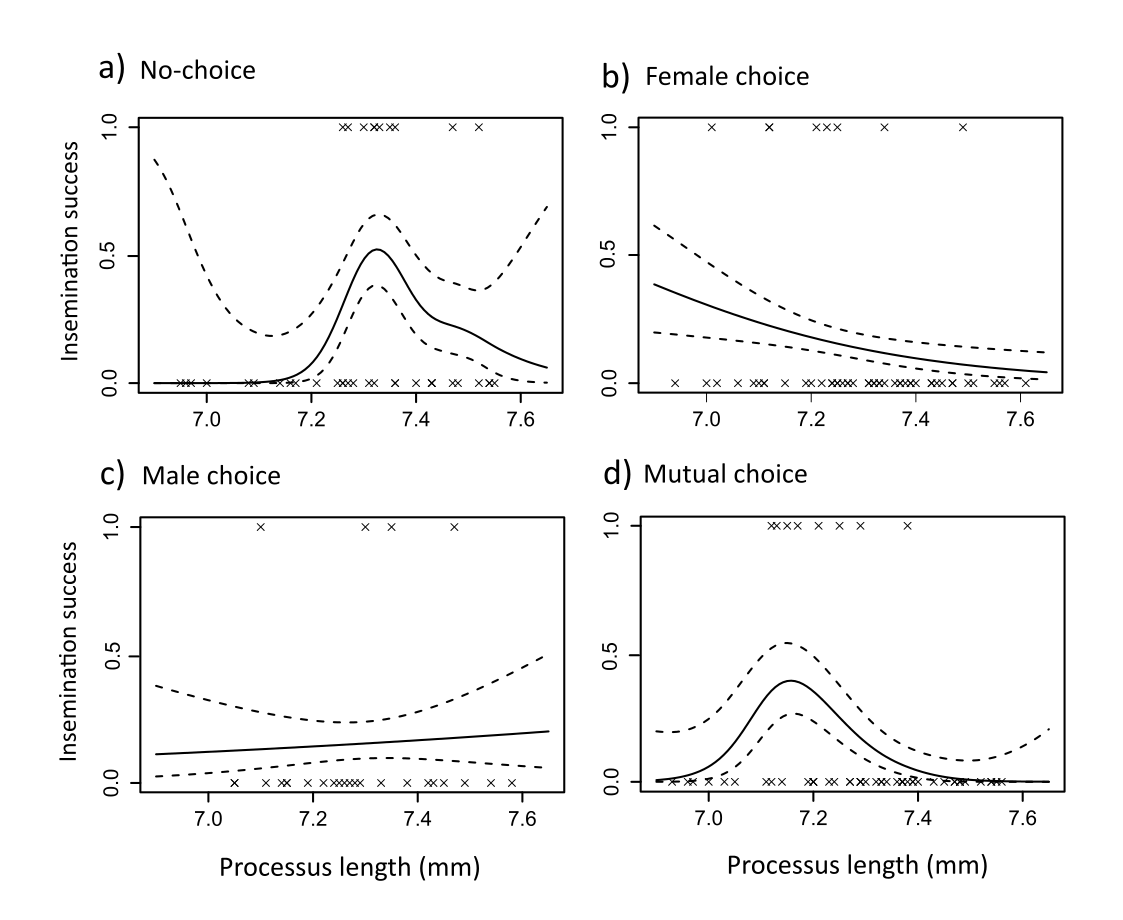


**Figure S1**. Fitness functions showing overall post-copulatory selection (using insemination success as the response variable, for all males including those who failed to mate) on male processus length in *L. equestris* for the four experimental choice treatments: a) No-choice (*N*= 39); b) Female choice (*N*= 54); c) Male choice (*N*= 26); and d) Mutual choice (*N*= 55). Dashed lines indicate 1 standard error above and below the predicted line.
